# Baicalin Protects ARPE-19 Cells Against BRVO-Related Hypoxic Injury by Preserving Mitochondrial Function and Inhibiting Ferroptosis

**DOI:** 10.3390/antiox15080934

**Published:** 2026-07-28

**Authors:** Yuchang Yang, Gaiyue Yue, Jie Bai, Jingwen Yang, Ziheng Wang, Qi Chen, Shuchang Yao, Lisha Yi, Xinzhu Wang, Jian Zhou, Jingyi Gao, Yaxuan Li, Ting Huang, Jian Ni, Changhai Qu

**Affiliations:** 1 College of Chinese Herbal Medicine, Beijing University of Chinese Medicine, Beijing 102488, China; 2 School of Chinese Medicine, Beijing University of Chinese Medicine, Beijing 100029, China

**Keywords:** baicalin, ARPE-19, branch retinal vein occlusion, ferroptosis, antioxidant

## Abstract

Branch retinal vein occlusion (BRVO) is a retinal vascular disorder characterized by ischemia and hypoxia. These pathological conditions contribute to retinal pigment epithelial (RPE) cell injury through oxidative stress and ferroptosis. However, whether baicalin (BC) protects against hypoxia-induced RPE injury remains unclear. CoCl_2_-induced hypoxic ARPE-19 cells were used to evaluate the protective effects and potential mechanisms of BC. BC improved cell viability and reduced LDH release under hypoxic conditions. BC markedly suppressed hypoxia-induced inflammatory responses, as evidenced by reduced p65 and ICAM-1 expression and decreased release of pro-inflammatory cytokines. Moreover, BC alleviated oxidative stress by reducing ROS and MDA accumulation and restoring SOD and GSH activity. BC attenuated mitochondrial dysfunction, accompanied by restoration of mitochondrial membrane potential, oxygen consumption rate (OCR), and ATP production. Transmission electron microscopy (TEM) results further confirmed the protective effect of BC on mitochondrial integrity. Mechanistically, BC suppressed ferroptosis by reducing intracellular Fe^2+^ accumulation and lipid peroxidation, accompanied by downregulation of TFR1, ACSL4, and p53, as well as upregulation of SLC7A11 and GPX4. These findings suggest that BC protects ARPE-19 cells against hypoxia-induced injury through preservation of mitochondrial function and inhibition of ferroptosis.

## 1. Introduction

Branch retinal vein occlusion (BRVO) is a common retinal vascular disorder characterized by localized ischemic hypoxia [1,2]. The retinal pigment epithelium (RPE), which supports photoreceptor function and maintains the blood–retinal barrier, is highly susceptible to hypoxic stress [3,4]. During BRVO, oxygen deprivation induces pathological alterations in RPE cells, ultimately resulting in barrier dysfunction and cell injury [5,6]. Because RPE integrity is essential for outer retinal homeostasis and barrier function, RPE dysfunction under ischemic stress may exacerbate secondary degeneration and compromise visual recovery [7,8]. Therefore, alleviating hypoxia-induced RPE injury may represent an important strategy for preserving visual function following retinal ischemia.

Hypoxia triggers profound intracellular metabolic reprogramming and oxidative stress. Excessive accumulation of reactive oxygen species (ROS) compromises mitochondrial bioenergetics and disrupts redox homeostasis, thereby contributing to cellular injury [9,10,11]. Notably, retinal pigment epithelial (RPE) cells continuously phagocytose shed photoreceptor outer segments, creating an intracellular milieu enriched in polyunsaturated fatty acids (PUFAs) [12,13]. This lipid-rich physiological feature renders RPE cells intrinsically susceptible to lipid peroxidation when redox balance is perturbed [14]. When antioxidant defenses fail, this PUFA-rich environment predisposes RPE cells to uncontrolled lipid peroxidation [15]. Such lipid toxicity ultimately culminates in ferroptosis, an iron-dependent, non-apoptotic form of cell death that is increasingly recognized as a key driver of hypoxic retinal injury [16,17]. Under hypoxic conditions, dysregulation of the HIF-1α/NRF2 axis and the SLC7A11/GPX4 antioxidant system may facilitate ferroptosis by amplifying oxidative stress and promoting iron accumulation [18]. Therefore, therapeutically targeting oxidative stress and ferroptosis may represent a promising strategy for hypoxia-induced retinal injury.

In recent years, traditional Chinese medicine (TCM) has drawn considerable attention for its potential role in the prevention and treatment of BRVO [19,20]. Baicalin (BC), a major flavonoid glycoside isolated from the roots of *Scutellaria baicalensis* Georgi, exhibits potent anti-inflammatory and antioxidant activities [21]. Recent studies have reported that BC alleviates retinal edema and helps maintain retinal structural integrity in RVO models, possibly by improving microcirculation and modulating VEGF expression [19]. In addition, BC has been linked to ferroptosis suppression in multiple disease settings, and this mechanism may contribute to its protective effects, including in macular degeneration [22]. Although these findings suggest a potential anti-ferroptotic role for BC in RPE cells, its capacity to rescue these cells from hypoxia-induced mitochondrial dysfunction and ferroptosis remains unexplored.

In this study, we used a CoCl_2_-induced hypoxic ARPE-19 model and found that baicalin mitigates hypoxia-related injury in vitro. This protection is associated with preserved mitochondrial function and reduced ferroptosis, at least in part through the SLC7A11/GPX4 axis. These results clarify a plausible mechanism by which baicalin protects RPE cells under hypoxic stress and support its further evaluation as a candidate therapy for ischemic retinal diseases, such as BRVO.

## 2. Materials and Methods

### 2.1. Reagents and Chemicals

Baicalin (purity > 98%) was obtained from Shanghai Yuanye Bio-Technology Co., Ltd. (Shanghai, China). Fetal bovine serum (FBS), 0.05% trypsin, Dulbecco’s Modified Eagle Medium: Nutrient Mixture F-12 (DMEM/F-12), DMEM, and penicillin/streptomycin solutions were purchased from Gibco, Invitrogen (Carlsbad, CA, USA). Phosphate-buffered saline (PBS) and 3-(4,5-dimethyl thiazol-2-yl-)-2,5-diphenyl tetrazolium bromide (MTT) were obtained from Beijing Solarbio Science and Technology Co., Ltd. (Beijing, China). ROS, MDA, GSH and SOD detection kits were purchased from Beyotime Biotechnology (Shanghai, China). The ELISA kits for IL-6, MCP1, IL-18, IL-1β and TNF-α were purchased from Biorigin (Beijing) Inc. (Beijing, China). The primary antibodies against NF-κB, ICAM-1, NRF2, HIF-1α, GPX4, P53, SLC7A11, ACSL4, TFR1 and β-actin were all purchased from Abcam (Cambridge, UK).

### 2.2. Cell Cultures and Treatment

The procedures for ARPE-19 cell culture and CoCl_2_-induced hypoxia modeling have been described in detail in our previous study [23]. In the present work, DMOG (300 μM) for 48 h was additionally employed alongside CoCl_2_ (500 μM) for 24 h to establish the in vitro hypoxia models. For drug intervention, BC dissolved in DMSO was administered to the cells. Based on the non-toxic threshold established in our prior research [23], the final vehicle concentration of DMSO was rigorously kept below 0.1% across all experimental groups.

### 2.3. Cell Viability Assay and Morphology Examination

Cell viability was assessed using the MTT assay according to our previous report [23].

### 2.4. Transmission Electron Microscopy (TEM)

Harvested ARPE-19 cells were primarily fixed in 2.5% glutaraldehyde (Beijing Kaiyue Biotechnology Co., Ltd., Beijing, China; Cat. No. K0012) overnight at 4 °C. After post-fixation with 1% osmium tetroxide (Ted Pella, Inc., Redding, CA, USA; Cat. No. 18456) and en bloc staining using uranyl acetate (Electron Microscopy Sciences, Hatfield, PA, USA; Cat. No. 22400), samples were dehydrated through graded ethanol (Sinopharm Chemical Reagent Co., Ltd., Shanghai, China; Cat. No. 10009218) and embedded in EPON resin (SPI Supplies, West Chester, PA, USA; Cat. No. 02659-AB) at 60 °C for 48 h. Ultrathin sections (60–80 nm) were prepared on copper grids and analyzed via a transmission electron microscope to evaluate mitochondrial ultrastructure, with a specific focus on mitochondrial swelling, cristae fragmentation, and outer membrane integrity.

### 2.5. MitoSOX Red Staining

To quantify superoxide generation, ARPE-19 cells were loaded with 5 μM MitoSOX Red indicator (10 min, 37 °C). Following warm PBS washes, the fluorescent signals were acquired and analyzed utilizing fluorescence microscopy.

### 2.6. Measurement of ROS, GSH, MDA and SOD Levels

As detailed in our previous publication [23], oxidative stress indicators including intracellular ROS, GSH, MDA, and SOD were measured using commercial kits in accordance with the manufacturers’ protocols. Intracellular ROS levels were tracked using a diluted DCFH-DA probe, while the levels of GSH, MDA, and SOD were analyzed using respective specific assay kits.

### 2.7. Assessment of Mitochondrial Function

Mitochondrial membrane potential (ΔΨm) was evaluated using JC-1 staining. Briefly, harvested ARPE-19 cells were incubated with JC-1 working solution for 20 min at 37 °C, washed with PBS, and analyzed by flow cytometry.

Cellular respiratory capacity was assessed by measuring the oxygen consumption rate (OCR) using an Enhanced Oxygen Consumption Rate Fluorometric Assay Kit. After adding the fluorescent probe according to the manufacturer’s instructions, fluorescence was recorded kinetically using a fluorescence microplate reader.

Intracellular ATP levels were quantified using an ATP Chemiluminescence Assay Kit. After treatment, cells were lysed and incubated with the detection reagent, and chemiluminescence was measured using a microplate reader. ATP signals were normalized to total protein content.

### 2.8. Measurement of Lipid Peroxidation and Intracellular Iron

Lipid peroxidation was determined using the BODIPY™ 581/591 C11 probe (Beyotime Biotechnology, Shanghai, China; Cat. No. S0043S). Harvested ARPE-19 cells were incubated with the probe for 30 min at 37 °C, washed, and analyzed by flow cytometry.

Intracellular ferrous iron (Fe^2+^) accumulation was measured using an Intracellular Iron (Fe^2+^) Assay Kit. Following treatment, cells were lysed and mixed with the kit reagents according to the manufacturer’s protocol. Absorbance was read on a microplate reader, and the results were normalized to total protein concentration.

### 2.9. Western Blot

Western blot analysis was carried out in accordance with our previously described methodology [23]. Following protein transfer and blocking, the PVDF membranes were probed with the indicated primary antibodies overnight at 4 °C, and subsequently incubated with the corresponding secondary antibodies for 2 h at room temperature. Protein signals were developed using a chemiluminescence reagent and the relative protein expression was quantified with ImageJ (version 1.54f; National Institutes of Health, Bethesda, MD, USA).

### 2.10. Quantitative Real-Time Polymerase Chain Reaction Analysis

Total RNA extraction and RT-qPCR analysis were performed as previously described. Briefly, ARPE-19 cells were treated as indicated, followed by total RNA isolation using TRIzol reagent (Thermo Fisher Scientific, Waltham, MA, USA). Primer sequences are listed in Table S1.

### 2.11. Statistics

Experiments were performed independently at least three times, and the results are expressed as mean ± standard deviation (SD). Statistical analysis was performed using GraphPad Prism 9.5 (GraphPad Software, LLC, Boston, MA, USA). Statistical significance was determined using one-way ANOVA followed by Tukey’s multiple comparison test. Differences were considered statistically significant at * *p* < 0.05, ** *p* < 0.01, and *** *p* < 0.001. Information on the software and instruments used is provided in Table S2.

## 3. Results

### 3.1. BC Attenuated Hypoxia-Induced Cytotoxicity In Vitro

**Figure 1:**
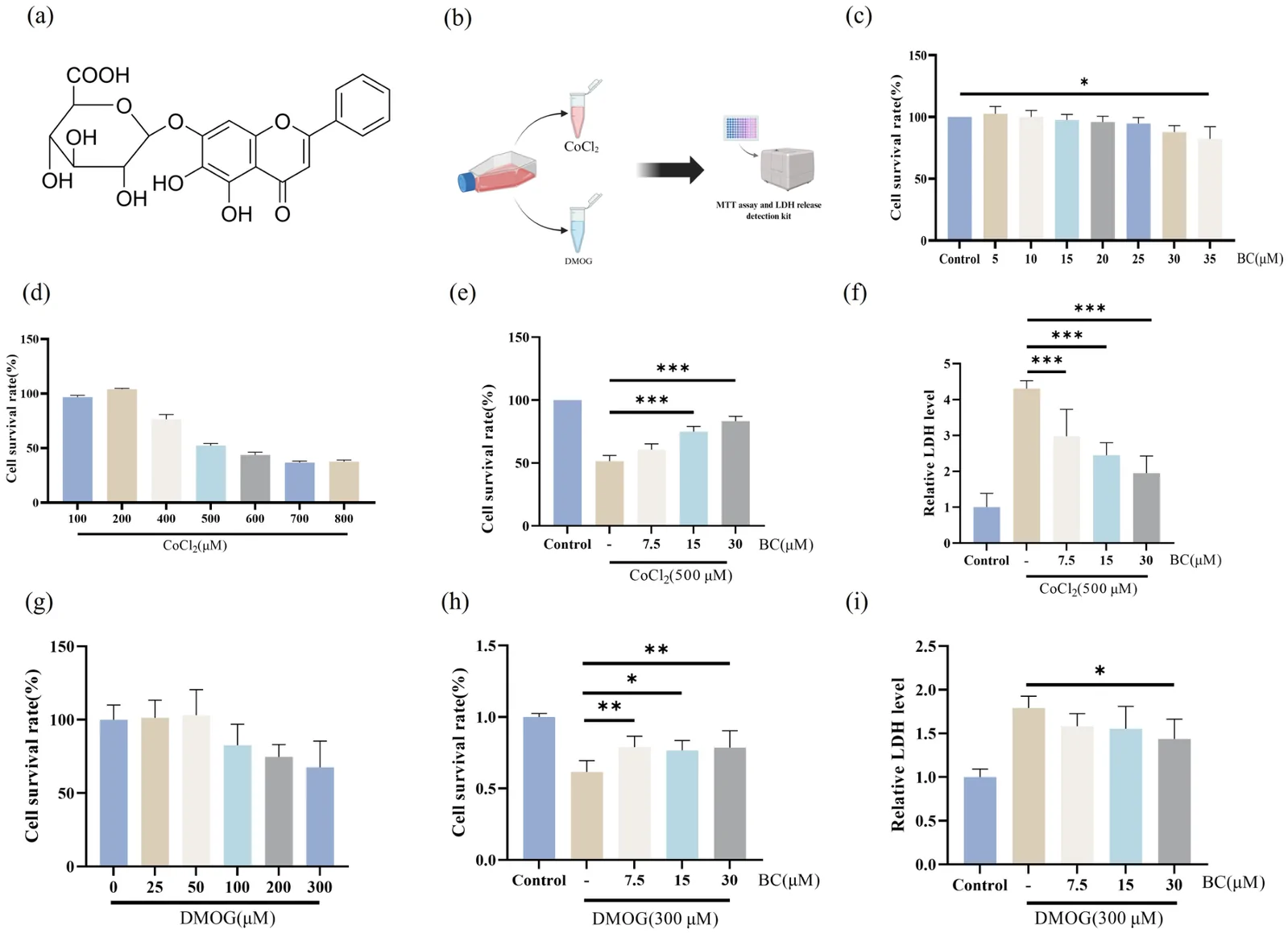
Protective effects of BC against CoCl_2_- and DMOG-induced injury in ARPE-19 cells. (**a**) Chemical structure of BC. (**b**) Schematic diagram of the experimental procedure. (**c**) Cell viability of ARPE-19 cells treated with various concentrations of BC to determine safe dosage limits. (**d**) Cell viability following exposure to different concentrations of CoCl_2_ (100–800 μM). (**e**) Effect of BC (7.5, 15, and 30 μM) on cell viability in the CoCl_2_-induced (500 μM) injury model, assessed by MTT assay. (**f**) Relative LDH release in the CoCl_2_ model with or without BC treatment. (**g**) Cell viability following exposure to different concentrations of DMOG (25–300 μM). (**h**) Effect of BC on cell viability in the DMOG-induced injury model, assessed by MTT assay. (**i**) Relative LDH release in the DMOG model with or without BC treatment. Data are presented as the mean ± SD (*n* = 3). * *p* < 0.05, ** *p* < 0.01, *** *p* < 0.001 vs. the model group. Different colors are used solely for visual distinction and do not represent specific groups or carry any additional meaning.

Figure 1a presents the chemical structure of BC, and the experimental protocol is shown in Figure 1b. MTT assay results demonstrated that BC at concentrations of 5–30 μM did not affect cell viability, whereas treatment with 35 μM BC significantly reduced cell viability (Figure 1c). Therefore, 30 μM was selected as the maximum concentration for subsequent experiments. CoCl_2_ exposure reduced ARPE-19 cell viability in a concentration-dependent manner. Treatment with 500 μM CoCl_2_ reduced cell viability to approximately 50% relative to untreated cells and was therefore selected to establish the hypoxic injury model in subsequent experiments (Figure 1d). Under CoCl_2_ stimulation (500 μM), BC treatment at 15 and 30 μM increased cell viability compared with the model group (Figure 1e). In addition, BC at 7.5, 15, and 30 μM reduced LDH release (Figure 1f). Cell viability gradually decreased following DMOG treatment (Figure 1g). In the DMOG-induced model, BC treatment at 7.5, 15, and 30 μM increased cell viability relative to the model group (Figure 1h). Moreover, 30 μM BC reduced LDH release compared with the model group (Figure 1i).

### 3.2. BC Suppressed CoCl_2_-Induced Inflammatory Responses in ARPE-19 Cells

**Figure 2:**
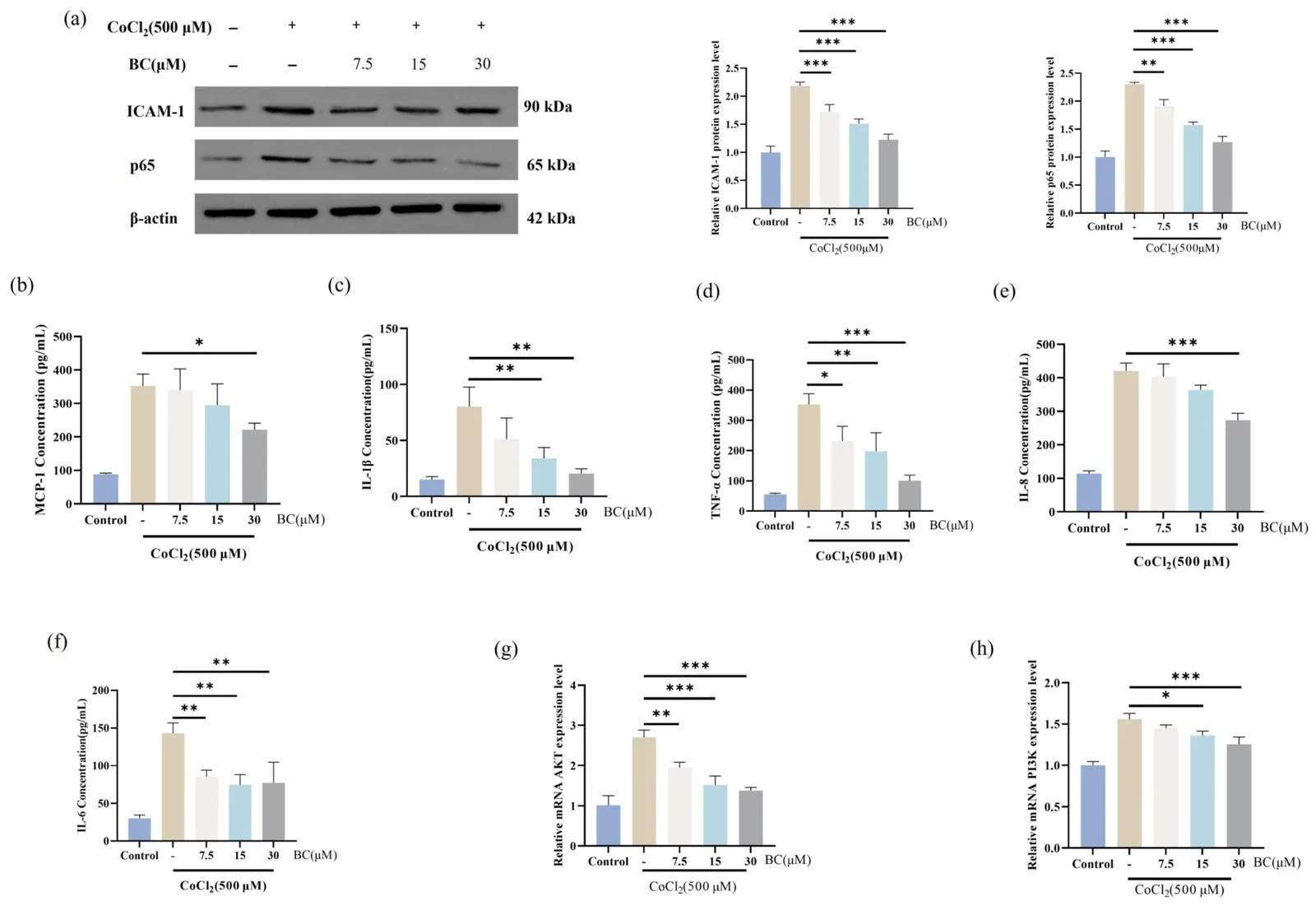
BC attenuates CoCl_2_-induced inflammatory responses in ARPE-19 cells. (**a**) Protein expression levels of p65 and ICAM-1 in ARPE-19 cells, as determined by Western blot analysis. (**b**–**f**) The release of inflammatory mediators, including MCP-1 (**b**), IL-1β (**c**), TNF-α (**d**), IL-18 (**e**), and IL-6 (**f**) in the cell culture supernatants, measured by ELISA. (**g**,**h**) Relative mRNA expression levels of PI3K (**g**) and AKT (**h**), assessed by RT-qPCR. Data are presented as the mean ± SD (*n* = 3). * *p* < 0.05, ** *p* < 0.01, *** *p* < 0.001 vs. the model group. Different colors are used solely for visual distinction and do not represent specific groups or carry any additional meaning.

The CoCl_2_-induced hypoxia model was used for subsequent experiments. Western blot analysis showed that Cocl_2_ exposure increased the protein expression levels of p65 and ICAM-1 in ARPE-19 cells compared with the control group, whereas BC treatment reduced their expression levels (Figure 2a). In parallel, ELISA results demonstrated that Cocl_2_ stimulation increased the levels of MCP-1, IL-1β, TNF-α, IL-18, and IL-6, while BC treatment decreased the release of these inflammatory mediators (Figure 2b–f), suggesting that BC attenuated hypoxia-induced inflammatory responses in ARPE-19 cells. In addition, PCR analysis showed that BC intervention reversed the Cocl_2_-induced alterations in PI3K and AKT mRNA expression (Figure 2g,h).

### 3.3. BC Alleviates CoCl_2_-Induced Metabolic Disturbances and Oxidative Stress in ARPE-19 Cells

**Figure 3:**
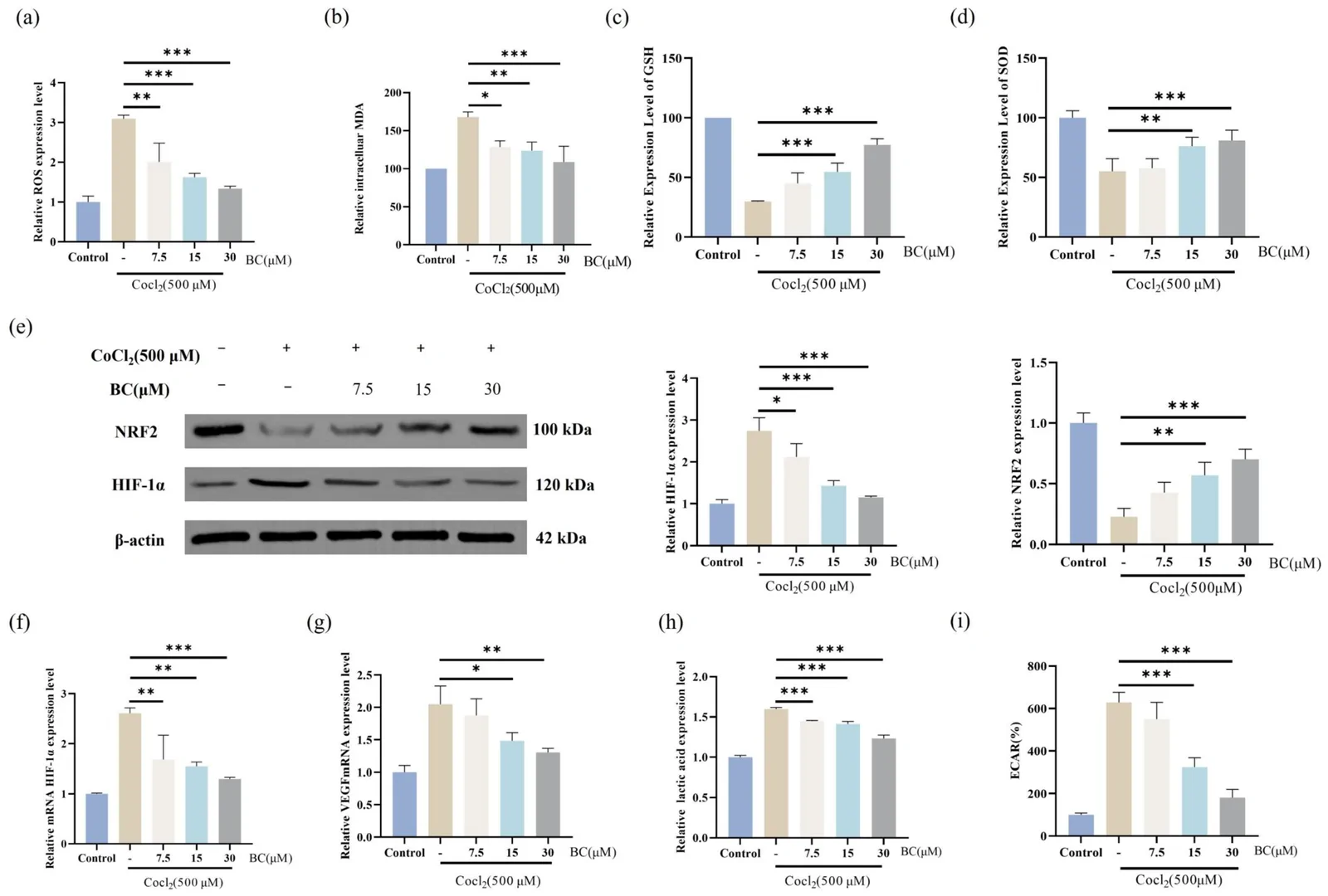
Effects of BC on CoCl_2_-induced oxidative stress and metabolic alterations in ARPE-19 cells. (**a**) Intracellular ROS levels in ARPE-19 cells (**b**–**d**) Levels of oxidative stress-related biochemical parameters, including MDA (**b**), GSH (**c**), and SOD (**d**). (**e**) Protein expression levels of NRF2 and HIF-1α, as determined by Western blot analysis. (**f**,**g**) Relative mRNA expression levels of HIF-1α (**f**) and VEGF (**g**), assessed by RT-qPCR. (**h**) Lactic acid production levels in ARPE-19 cells. (**i**) ECAR representing the glycolytic capacity. Data are presented as the mean ± SD (*n* = 3). * *p* < 0.05, ** *p* < 0.01, *** *p* < 0.001 vs. the model group. Different colors are used solely for visual distinction and do not represent specific groups or carry any additional meaning.

CoCl_2_ exposure induced pronounced hypoxia-associated oxidative stress and metabolic alterations in ARPE-19 cells. As shown in Figure 3a–d, intracellular ROS and MDA levels were markedly elevated following CoCl_2_ stimulation, whereas the antioxidant-related indices GSH and SOD were significantly decreased, indicating disruption of cellular redox homeostasis. Consistently, Western blot analysis showed that CoCl_2_ exposure markedly increased HIF-1α accumulation, accompanied by disruption of the NRF2-mediated antioxidant response, whereas BC treatment largely reversed these alterations (Figure 3e). Since HIF-1α is a key regulator of hypoxia-adaptive signaling, its downstream transcriptional activity was further evaluated [24]. RT-qPCR analysis demonstrated that CoCl2 treatment significantly upregulated HIF-1α mRNA and its target gene VEGF, while BC administration effectively suppressed these changes (Figure 3f,g), indicating that BC attenuated hypoxia-responsive signaling under oxidative stress conditions. Considering that hypoxia may affect cellular metabolism, lactate production and Extracellular acidification rate (ECAR) were further evaluated in ARPE-19 cells [25]. CoCl_2_ treatment significantly increased both lactate levels and ECAR (Figure 3h,i), accompanied by excessive ROS accumulation and impaired antioxidant capacity.

### 3.4. BC Alleviates CoCl_2_-Induced Mitochondrial Dysfunction in ARPE-19 Cells

**Figure 4:**
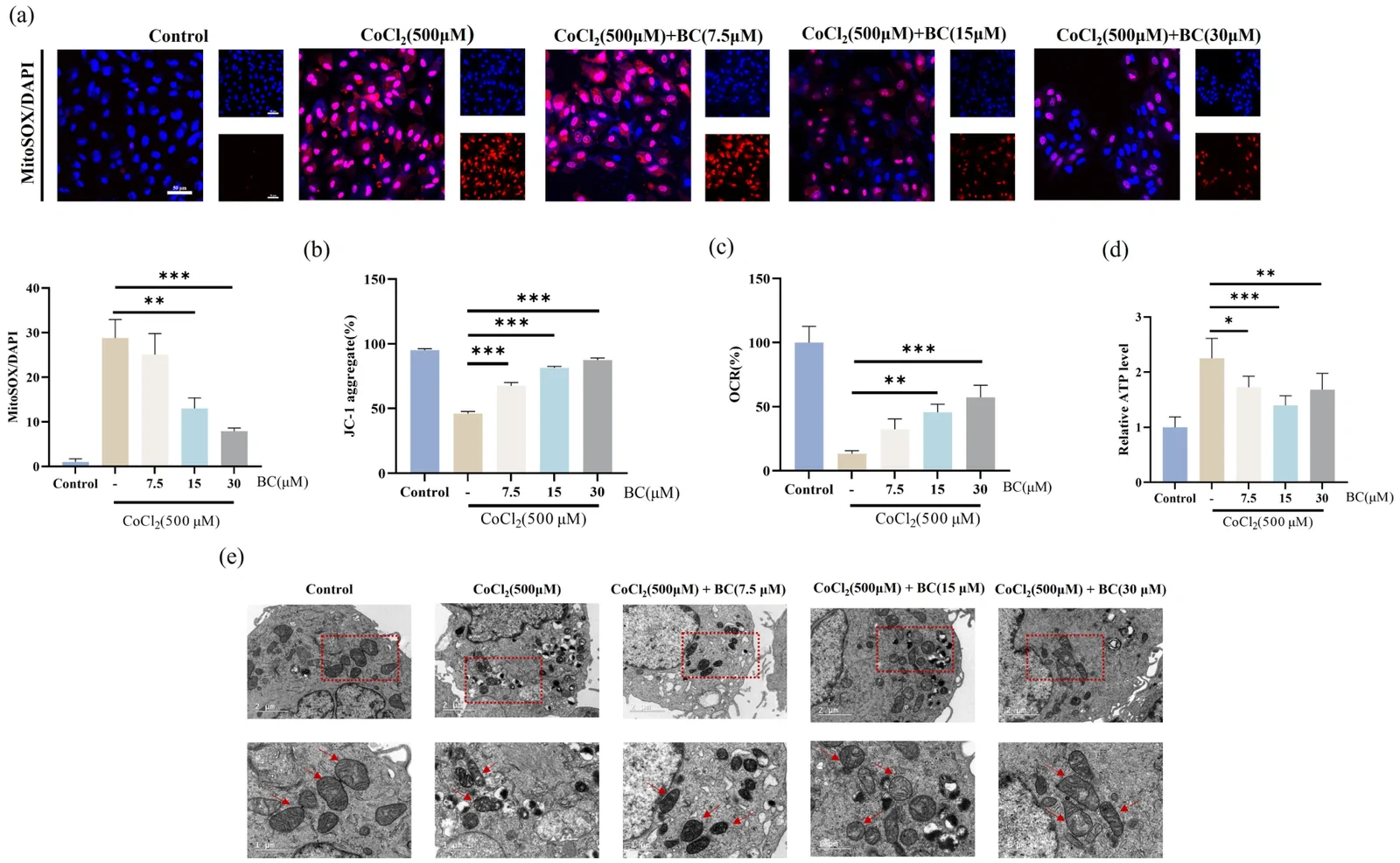
BC alleviates CoCl_2_-induced mitochondrial dysfunction and structural damage in ARPE-19 cells. (**a**) Measurement of mitochondrial ROS accumulation. (**b**) Evaluation of mitochondrial membrane potential using JC-1 staining. (**c**) OCR, representing mitochondrial respiratory capacity. (**d**) Measurement of intracellular ATP levels. (**e**) Representative TEM images showing mitochondrial ultrastructural changes (red arrow, mitochondrion). Data are presented as the mean ± SD (*n* = 3). * *p* < 0.05, ** *p* < 0.01, *** *p* < 0.001 vs. the model group. Different colors are used solely for visual distinction and do not represent specific groups or carry any additional meaning.

CoCl_2_ exposure disrupted mitochondrial bioenergetics and homeostasis in ARPE-19 cells. Specifically, the model group exhibited elevated mitochondrial ROS accumulation (Figure 4a) and a consequent loss of mitochondrial membrane potential, as indicated by JC-1 staining (Figure 4b). These mitochondrial abnormalities further impaired respiratory function, evidenced by a decreased OCR (Figure 4c) and depleted intracellular ATP levels (Figure 4d). BC treatment effectively reversed these functional deficits, suppressing ROS generation, restoring membrane potential, and recovering both OCR and ATP production. To correlate these metabolic impairments with physical alterations, mitochondrial ultrastructure was evaluated via TEM. CoCl_2_ exposure induced morphological damage, including swelling, cristae fragmentation, and vacuolar degeneration. Importantly, these ultrastructural aberrations are classic morphological hallmarks of ferroptosis. BC intervention preserved mitochondrial architecture and attenuated these degenerative changes (Figure 4e).

### 3.5. BC Mitigates CoCl_2_-Induced ARPE-19 Cell Death by Suppressing Ferroptosis

**Figure 5:**
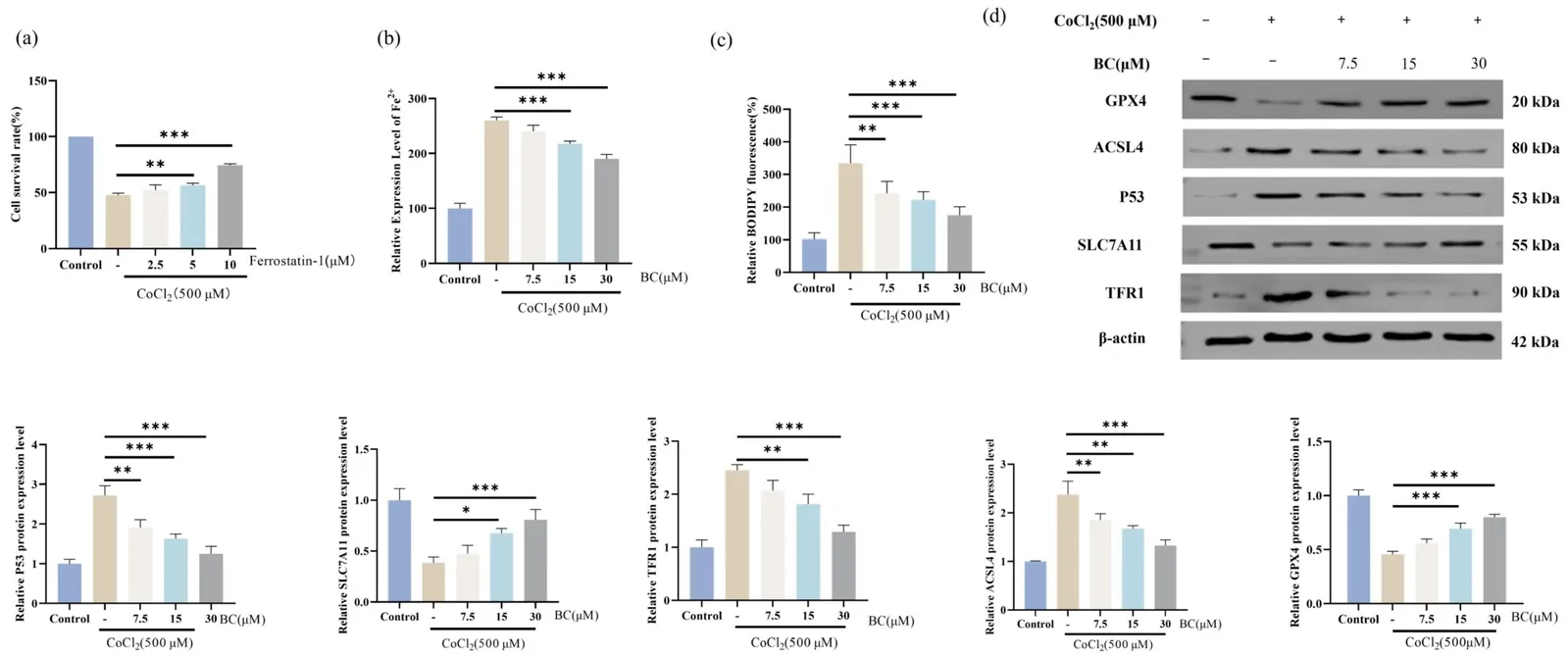
BC mitigates CoCl_2_-induced ferroptosis in ARPE-19 cells. (**a**) Cell viability assessed by MTT assay following treatment with the specific ferroptosis inhibitor Fer-1. (**b**) Measurement of intracellular iron (Fe^2+^) levels. (**c**) Evaluation of lipid peroxidation accumulation using BODIPY staining. (**d**) Protein expression levels of ferroptosis-related regulators, including TFR1, ACSL4, p53, SLC7A11, and GPX4, as determined by Western blot analysis. Data are presented as the mean ± SD (*n* = 3). * *p* < 0.05, ** *p* < 0.01, *** *p* < 0.001 vs. the model group. Different colors are used solely for visual distinction and do not represent specific groups or carry any additional meaning.

The mitochondrial ultrastructural alterations, particularly disrupted cristae and vacuolar degeneration, correspond to the morphological signatures of ferroptosis [26]. Given these structural changes and the observed ROS accumulation, we investigated whether ferroptosis mediates CoCl_2_-induced cell death. To test this, the specific ferroptosis inhibitor Ferrostatin-1 (Fer-1) was introduced. MTT assay showed that Fer-1 treatment rescued the viability of CoCl_2_-exposed ARPE-19 cells, confirming the involvement of ferroptotic cell death in this model (Figure 5a). Biochemically, ferroptosis is characterized by iron accumulation and lipid peroxidation. CoCl_2_ exposure induced intracellular iron overload, indicated by increased Fe^2+^ levels (Figure 5b), alongside enhanced lipid peroxidation as detected by BODIPY staining (Figure 5c). BC treatment attenuated these biochemical alterations, decreasing intracellular Fe^2+^ levels and reducing lipid peroxidation. To define the molecular basis of this anti-ferroptotic effect, core regulatory proteins were assessed. Western blot analysis revealed that CoCl_2_ exposure upregulated pro-ferroptotic factors, including TFR1, ACSL4, and p53, while downregulating components of the antioxidant defense system, SLC7A11 and GPX4. BC treatment reversed these molecular alterations, decreasing TFR1, ACSL4, and p53 expression, and upregulating the SLC7A11/GPX4 axis (Figure 5d).

## 4. Discussion

BRVO is characterized by localized retinal ischemia and oxygen deprivation, which contribute to progressive retinal degeneration [27]. As a critical component of the outer blood–retinal barrier, the RPE is particularly vulnerable to ischemic stress [28]. To model hypoxic injury in vitro, ARPE-19 cells were treated with CoCl2, which stabilizes hypoxia-related signaling and promotes intracellular ROS accumulation [29,30,31]. BC improved cell viability and reduced LDH release in CoCl_2_-treated ARPE-19 cells, indicating that it preserved cellular viability and alleviated plasma membrane damage under hypoxia-mimetic conditions. BC also reduced ROS accumulation, restored antioxidant capacity, and modulated HIF-1α/NRF2-related responses. These findings suggest that the cytoprotective effect of BC is associated with the restoration of redox homeostasis and the attenuation of hypoxia-induced oxidative stress and metabolic disturbance. Similar protective effects were observed in the DMOG-induced injury model, supporting the interpretation that the effect of BC was not limited to CoCl_2_-specific cytotoxicity but was also associated with cellular responses commonly induced by hypoxia-mimetic stress.

Oxidative stress and inflammation are critical pathological events within the hypoxic retinal microenvironment [32,33]. Our findings showed that CoCl_2_ exposure promoted pathological HIF-1α accumulation and VEGF transcriptional activation while disrupting NRF2-mediated antioxidant defense, leading to excessive ROS generation and redox imbalance. Sustained oxidative stress may impair the barrier and metabolic functions of RPE cells and amplify local inflammatory responses, thereby contributing to the progression of ischemic retinal injury in BRVO. The resulting oxidative stress further activated inflammatory signaling pathways, as evidenced by increased p65 and ICAM-1 expression and elevated secretion of pro-inflammatory cytokines, including MCP-1, IL-1β, and TNF-α. Additionally, PI3K and AKT transcription was upregulated by CoCl_2_ and reversed by BC. ROS can activate PI3K/AKT signaling through oxidative inhibition of phosphatases such as PTEN [34]. Given that BC restored NRF2 activity in our study, its anti-inflammatory effects may be partly attributed to reduced oxidative stress and subsequent suppression of PI3K/AKT-mediated inflammatory signaling. However, this proposed mechanism requires further validation at the protein phosphorylation level.

The RPE, which maintains retinal homeostasis and the blood–retinal barrier, is highly susceptible to hypoxic stress [35,36]. In the present study, CoCl_2_ exposure increased lactate production and ECAR in ARPE-19 cells, suggesting enhanced glycolytic activity under hypoxic conditions. These metabolic alterations were accompanied by mitochondrial dysfunction, including reduced OCR and ATP production. BC treatment partially reversed these changes and restored mitochondrial membrane potential and respiratory function, indicating that preservation of mitochondrial homeostasis may contribute to its protective effects against hypoxic injury [37]. The maintenance of RPE metabolic function is particularly important because these cells provide essential nutritional and metabolic support to adjacent photoreceptors. Therefore, prolonged hypoxia-induced metabolic dysfunction in the RPE may compromise photoreceptor homeostasis and contribute to retinal functional impairment in BRVO [38].

Mitochondria are central regulators of both cellular bioenergetics and cell fate determination [39,40]. TEM analysis revealed severe mitochondrial abnormalities under hypoxic conditions, including swelling, cristae fragmentation, and vacuolar degeneration. Notably, these ultrastructural changes are characteristic morphological features of ferroptosis [41,42]. Treatment with the ferroptosis inhibitor Ferrostatin-1 partially rescued hypoxia-induced cell death, supporting the involvement of ferroptosis in hypoxic injury. Mechanistically, hypoxia induced intracellular Fe^2+^ accumulation and lipid peroxidation, along with dysregulation of ferroptosis-related proteins. BC reversed this iron-dependent lipid peroxidation and the associated protein dysregulation, suggesting that inhibition of ferroptosis may contribute to its protective effects against hypoxia-induced RPE injury.

Several limitations should be acknowledged. Although ARPE-19 cells provide a practical model for studying RPE injury, they do not fully retain the phenotype and functional characteristics of native human RPE cells. Moreover, CoCl_2_- and DMOG-induced chemical hypoxia represents only part of the complex ischemic and inflammatory microenvironment present in BRVO. The present findings should therefore be interpreted within the context of this simplified in vitro model.

## 5. Conclusions

In conclusion, BC protected ARPE-19 cells against CoCl_2_-induced hypoxic injury in vitro. Mechanistically, BC alleviated hypoxia-induced mitochondrial dysfunction and suppressed ferroptosis through modulation of the SLC7A11/GPX4 axis, while also attenuating oxidative stress and inflammatory responses. These findings suggest that BC may serve as a potential therapeutic candidate for hypoxia-induced RPE injury in ischemic retinal diseases.

## Data Availability

The original contributions presented in the study are included in the article, further inquiries can be directed to the corresponding authors.

## Supplementary Materials

The following supporting information can be downloaded at: https://www.mdpi.com/article/10.3390/antiox15080934/s1, Table S1: RT-qPCR primer sequences; Table S2: Instruments and analytical software.
