# Supplementary material for: Baicalin Protects ARPE-19 Cells Against BRVO-Related Hypoxic Injury by Preserving Mitochondrial Function and Inhibiting Ferroptosis
Source: Antioxidants (Basel). 2026 Jul 28;15(8):934. doi: 10.3390/antiox15080934 (PMC13509413; doi:10.3390/antiox15080934)
Supplement: Supplementary file 1 [file antioxidants-15-00934-s001.zip › antioxidants-4415030-supplementary.pdf]

Table S1. RT-qPCR primer sequences

Gene	Primer	Sequence (5'-3')
GAPDH	Forward	CAGGAGCGAGACCCCACTAA
	Reverse	ATCACGCCACAGCTTTCCAG
HIF-1α	Forward	CTCAGAATGAAGTGTACCCTAA
	Reverse	CAAATCAGCACCAAGCAG
VEGF	Forward	TCACCAAGGCCAGCACAT
	Reverse	GGCTCCAGGGCATTAGACA
AKT	Forward	GCGACGTGGCTATTGTGA
	Reverse	CAGTCTGGATGGCGGTTG
PI3K	Forward	AACAACCTTATTATTACCCTCC
	Reverse	CCTATCCTCCGATTACCA

Table S2. Instruments and analytical software.

Instrument/Software	Manufacturer/Developer & Location
Multiskan GO plate reader	Thermo Scientific, USA
IX71 inverted microscopy	Olympus, Japan
ImageJ software (version 1.54f)	National Institutes of Health, Bethesda, MD, USA
NovaSeq 6000 platform	Illumina (Sequenced by LC-Bio Technology, Hangzhou, China)
Transmission electron microscope	Hitachi, Japan
QuantStudio 5 Real-Time PCR instrument	Applied Biosystems, CA, USA
iQ5 Real-Time PCR System	Applied Biosystems, CA, USA
FACSCanto II flow cytometer	BD Biosciences, USA
FlowJo software (version 10.0)	FlowJo LLC, Ashland, OR, USA
